# Safety of a gastropexy device in infants and young children in percutaneous endoscopic gastrostomy tube placement

**DOI:** 10.1038/s41598-025-96077-5

**Published:** 2025-05-07

**Authors:** G. Gavallér, M. Bukva, P. Gál, P. Pásztor, A. T. Takács, Cs. Bereczki, D. Szűcs

**Affiliations:** 1 Department of Pediatrics and Pediatric Health Centre, University of Szeged, Szeged, Hungary; 2 Department of Immunology, University of Szeged, Szeged, Hungary; 3 Data Science and Me LLC, Kecskemét, Hungary

**Keywords:** Push gastrostomy, Gastropexy device, Infants, Enteral feeding, Gastroenterology, Paediatric research, Nutrition

## Abstract

In our practice, there is a growing need to perform gastrostomy tube placement in infants and young children with feeding difficulties. To avoid possible complications arising from pull-through method (pull-through PEG) we began to perform a one-step endoscopic gastrostomy with a gastropexy device (push GT). This study aimed to evaluate the safety of this technique in infants and young children. In our study, 60 pull-through PEG and push GT procedures were retrospectively analysed in patients between 2.83 and 8.6 kg. We analysed the adverse events in both groups. Age, sex, weight, diagnosis, early (occurring ≤ 7 days after the procedure) and late (occurring > 7 days after the procedure) complications were compared in the two groups. Median follow-up duration was 12 months. Early minor complications occurred only in the push GT group, but this was not statistically significant. There was no significant difference between the groups regarding early major complications. Late minor complications were significantly more common in the push GT group. There were no late major complications in the push GT group, which is statistically significant. In infants and young children, push GT with a gastropexy device is a safe method to perform gastrostomy even in patients unsuitable for pull-through PEG placement.

## Introduction

Percutaneous endoscopic gastrostomy (PEG) tube insertion to maintain adequate nutritional support has become a frequently performed intervention. The number of indications for inserting a feeding tube has increasing^1^. The most frequent indication is neurological impairment, but there are many other diseases, such as cystic fibrosis, inflammatory bowel diseases, congenital defects, nephrological, oncological, metabolic diseases or anatomical malformations where there are higher energy needs and/or reduced oral feeding, which requires supplementation^1^.

In our practice, the need for gastrostomy tube placement among infants and young children has increased in the past decade, probably because endoscopic procedures have made it more feasible.

The most preferred method is the classic pull-through technique, which was first described in 1979 by Gauderer and Ponsky^2^. This technique has replaced surgical gastrostomy to enteral nutrition delivery because it has important advantages compared to open or laparoscopic techniques^3,4^.

Later, in 1984, Russel et al. first described the push technique but without fixation of the stomach to the abdominal wall^5^.

Brown et al. first described the use of the T-bar technique with fluoroscopy in radiological literature in 1986^6^. Later, in 1996, Boswell et al. first described the use of T-bar fasteners in children to fix the stomach to the anterior abdominal wall^7^. In 2007, Terry et al. described the safety and efficacy of the push technique with T-fasteners in paediatric patients, including 15 infants younger than 1 year^8^. In 2008, the Mickey Introducer Kit became available, which allows for the initial placement of low-profile balloon G-tubes^9^.

Recently one-step button or push gastrostomy has become popular. It can spare repeated general anaesthesia for the removal of the tube and it is cost effective despite the higher initial cost^9–11^. “The one-step device is also preferable in patients with a higher anaesthetic risk, previous cardiac or oesophageal surgeries as the passage of the large bumper down the oesophagus is avoided.”^1^.

Studies in the literature confirm the safety of this method in infants^8–12^.

Analysis of the complications of the two methods concluded that one-step gastrostomy is associated with a low rate of local infections; however, infants have a higher risk^11^.

Late complications caused by T-fasteners are relevant and they are associated with significant morbidity in infants and premature babies^11,13^. Finding the right time to remove T-fasteners might be able to reduce this type of complications^14^.

In our tertiary care centre, we started to perform one-step or push gastrostomy tube placement with a gastropexy device in 2017. The gastropexy device was used for dual gastropexy. This method is well known in the literature and is used in adults to perform gastropexy^15–18^.

The initial use of a gastric tube is due to local financial reasons. Later the gastrostomy tube can be replaced with a gastrostomy button in alert patients. This study aimed to analyse the outcomes of conventional pull-through PEG and push GT under 10 kg focusing on underlying diseases, as well as early and late complication rates and types.

## Materials and methods

### Ethical approval

This study was conducted in accordance with the Declaration of Helsinki. The study protocol was approved by the Human Investigation Review Board of the University of Szeged, Albert Szent-Györgyi Clinical Centre. (Approval No. 118/2023-SZTE)

### Patients

Between 2013 and 2023 a total of 136 endoscopic device insertions (pull-through PEG and push GT) were performed in our tertiary paediatric gastroenterological centre excluding laparoscopic-assisted PEG insertions. From 2017 the decision to perform push GT was based first on patient characteristics. Infants and young children under 10 kg, patients with a history of oral, upper gastrointestinal, or respiratory tract surgery, the paediatric intensive care population, and patients expected to weigh less than 10 kg at the time of device replacement were eligible for gastropexy. To evaluate of the methodical shift from pull-through PEG to push GT under 10 kg we conducted a retrospective study.

Study selection criteria were patients under 10 kg who required a long-term enteral feeding route for the first time by performing a pull-through PEG or push GT. None of the patients have previous abdominal surgery, and all were eligible for percutaneous endoscopic gastrostomy. The indication for nutritional support was for different reasons. As a result of the weight limit, our study group included 60 patients.

### Collection of data

Both groups of patients were identified by an electronic search for the PEG procedure in our electronic medical programme. We analysed these two groups of patients with respect to age, sex, weight, underlying diseases, early and late, as well as minor and major complications. All patients were Caucasians.

Early complications occurred ≤ 7 days after the procedure and late complications occurred > 7 days after the procedure. Regarding minor and major complications, we used the categories of the European Society of Paediatric Gastroenterology, Hepatology and Nutrition guidelines^1^.

Stomal infection was defined by the need for systemic antibiotics. We did not examine non-gastrostomy-related complications (i.e., those directly linked to the underlying disease).

It should be noted that some of the complications are related to the surgical procedure, while others are due to the characteristics of the device. In our study we analysed all possible complications related to gastrostomy together.

The procedures were performed in the endoscopic room or by patients requiring intensive care in the intensive care unit. Duration of the procedure, perioperative complications were recorded, and concomitant procedure was not performed.

Pull-through PEG procedures require general anaesthesia and intubation of patients. The push GT procedure is performed under general anaesthesia but expect that for mechanically ventilated patients intubation is not necessary. In pull-through PEG group a broad-spectrum antibiotic was administered intravenously prior to the procedure, following the guidelines. In push GT group we maintain aseptic conditions during the procedure, but preoperative antibiotics are not used.

As postoperative care, we follow all patients. Enteral liquids were started 8 h after the procedure and tube feeding was started on the first postoperative day, if we had not observed any complications. The patients were discharged to their home or to their local hospital, as appropriate.

### Operative techniques

#### Original pull-through technique

The flexible gastroscope we used was Fujinon EG-530WR [outer diameter: 9.4 mm] or Fujinon EG-530 N [outer diameter: 5.9 mm]; Fujinon, Wayne, NJ, USA). PEG is a well-established procedure and we consider it essential to find an appropriate and safe site for puncture through gastric insufflation and transilluminating the abdomen. At the end of the procedure, the internal bumper and the external part of the tube fasten the stomach to the anterior wall. Parents were instructed to rotate the tube 360 daily after two weeks.

#### One-step percutaneous endoscopic gastrostomy

##### Push gastrostomy with gastropexy device

The flexible gastroscope that we used was Fujinon EG-530WR [outer diameter: 9.4 mm] or Fujinon EG-530 N [outer diameter: 5.9 mm]; Fujinon, Wayne, NJ, USA). We use Freka gaxtropexy device II (Fresubin Kabi AG) with introducer, dilatator (Vygon peel away desilet, introducer for 14 Fr catheter) and with Flocare 10 Ch gastrotube. The Freka gastropexy device II has two parallel needles and a suture-holding loop. After endoscopic transillumination we choose the right place for the two parallel gastropexy. We use the device according to the manufacturer’s instructions. At the end of the first gastropexy, we tie the two ends of the thread into a knot. In the same way, a second gastropexy is made about 1–1.5 cm away as space permitted. Next, a stoma channel is made using Seldinger technique with an introducer and dilatator in the centre of gastropexy. Under endoscopic control, we observe atraumatic insertion. In the next step, with a peel-away sheath technique, a 10 Ch gastric tube is inserted into the stoma and fixed by inflating the balloon. Suture threads are removed (cut) 14 days later in outpatient settings in our clinic or another hospital in case of discharge. Our operative team consists of a gastroenterologist and an intensive care specialist in one procedure.

##### One-step button with T- fasteners

This technique is performed under general anaesthesia and under endoscopic control. The first step in this technique is to do gastropexy. For this reason, 3 T-fasteners in trigonal shape are used to fix the stomach to the abdominal wall. These fasteners are preloaded gastrointestinal suture anchor systems that incorporate resorbable sutures and external suture locks. The anchors left in place until the suture resorb and slough with internal components passing through the gastrointestinal tract^11^. After gastropexy a stoma channel is made using a modified Seldinger technique and a dilatator in the centre of gastropexy. After dilatation a gastrostomy tube is inserted, and the balloon filled with water^13^. Dahlseng et al. made some modifications to insert a button and avoid too much dilatation. In the literature prophylactic antibiotic is given^14^.

### Statistical analysis

Descriptive statistics were used to summarize the clinical parameters of age, weight (in grams) and gender distribution in the push GT and pull-through PEG groups. The analysis included calculating the minimum, maximum, median, interquartile range (IQR), mean and standard deviation (SD) for continuous variables (age and weight).

To compare the distribution of age and weight between the push GT and pull-through PEG groups, the Wilcoxon rank sum test was used given the non-parametric nature of the data. A *p*-value of less than 0.05 was considered statistically significant. Confidence intervals were also calculated and presented alongside median differences.

Gender distribution between the push GT and pull-through PEG groups was analysed using Pearson’s Chi-squared test. This test assessed whether there was a significant difference in the proportion of males and females between the two groups.

For each underlying medical condition and further postoperative complications, the appropriate statistical test was selected based on the expected frequencies in the contingency tables. Pearson’s Chi-squared test was used when all expected frequencies were 5 or greater. If any expected frequency was less than 5, Fisher’s exact test was employed to ensure accuracy. Confidence intervals were not calculated, as this is not possible for multidimensional contingency table analysed by Pearson’s Chi-squared test.

## Results

A total of 60 patients were identified and analysed with respect to age, weight, sex, underlying diseases, early and late, as well as minor and major complications.

Median follow-up was 12 months for both groups. In the pull-through PEG group, we lost 1 patient during the follow-up. Seven patients in the pull-through PEG group and six patients in the push GT group died of their underlying disease during the first year after the procedure.

### Basic characteristics of the push GT and pull-through PEG patient groups

**Fig. 1:**
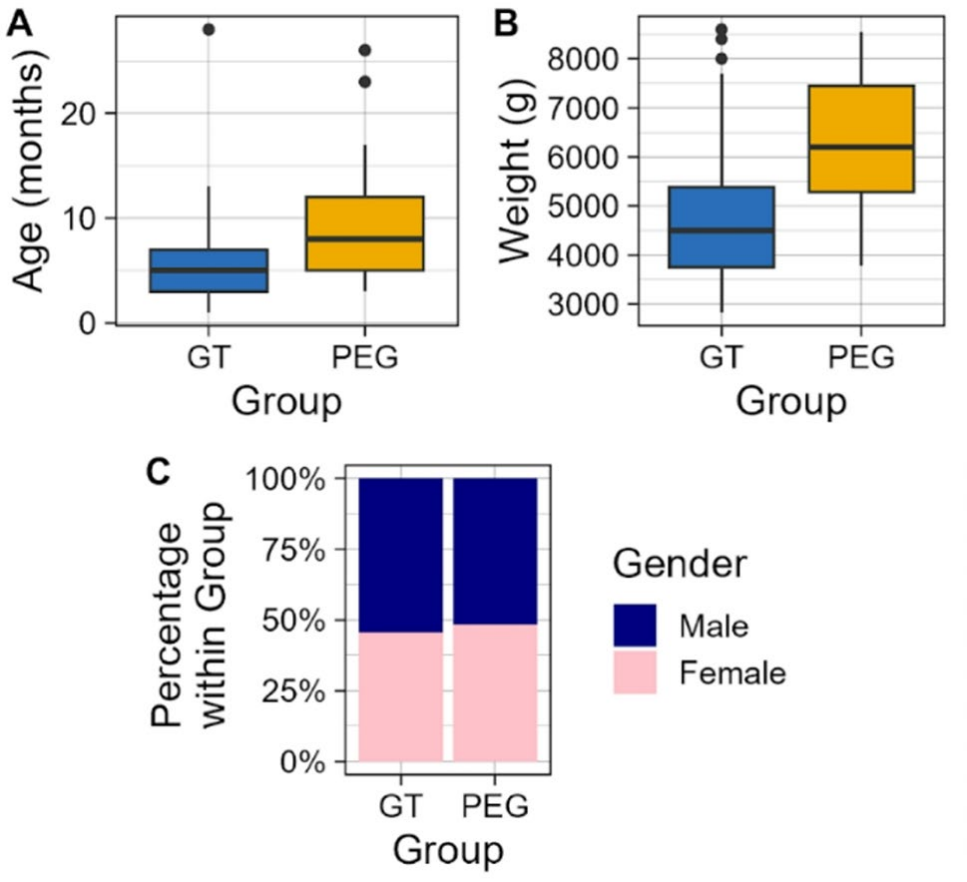
Basic clinical parameters of the push GT and pull-through PEG Groups. (**A**) The age distribution in months for the push GT and the pull-through PEG groups. The boxplots show the median (central line), the interquartile range (IQR) (box), and the minimum and maximum values (whiskers). It highlights the significant (*p* = 0.0007) difference in age between the two groups. (**B**) The weight distribution in grams for the push GT and pull-through PEG groups. The boxplots display the median (central line), the interquartile range (IQR) (box), and the minimum and maximum values (whiskers). This demonstrates a significant difference in weight between the groups (*p* = 0.0002). (**C**) The gender distribution within the push GT and pull-through PEG groups using bar charts, indicating that there is no significant difference in gender distribution between the two groups (*p* = 0.8352).

The basic clinical parameters of the push GT and pull-through PEG groups are summarized in Fig. 1. There was a significant difference in age at gastrostomy formation between the push GT and pull-through PEG groups (*p* = 0.0007, Wilcoxon rank sum test, 95% CI for median difference: 2.0–5.0 months) (Fig. 1A). The median age of the push GT group was 5.0 months (IQR: 3.0–7.0), ranging from 1.0 to 28.0 months. In contrast, the median age in the pull-through PEG group was 8.0 months (IQR: 5.0–12.0), ranging from 3.0 to 26.0 months.

Body weight (in grams) at the time of gastrostomy formation also showed a significant difference between the groups (*p* = 0.0002, Wilcoxon rank sum test, 95% CI for median difference: 1200–2000 g) (Fig. 1B). The push GT group had a median weight of 4500.0 g (IQR: 3750.0–5380.0), ranging from 2830.0 to 8600.0 g. The pull-through PEG group had a higher median weight of 6200.0 g (IQR: 5280.0–7450.0), ranging from 3780.0 to 8550.0 g.

In terms of gender distribution, there was no significant difference between the push GT and pull-through PEG groups (*p* = 0.8352, Pearson’s Chi-squared test) (Fig. 1C). In the push GT group, 18 participants (54.54%) were male and 15 (45.45%) were female. In the pull-through PEG group, 14 participants (51.85%) were male and 13 (48.14%) were female.

### Underlying medical conditions in the push GT and pull-through PEG groups

The analysis of underlying medical conditions in the push GT and pull-through PEG groups is summarized in Table 1. There were no significant differences between the two groups for most conditions, including neurological impairment, metabolic disorders, patients with a tracheostomy tube or in need of respiratory support, cardiological conditions, nephrological conditions and immunodeficiency. Patients in the group ‘Others’ had various disorders, such as cleft palate, ichthyosis (Netherton syndrome), intestinal lymphangiectasia, VACTERL association and myopathy. The occurrence of oncological conditions; however, showed a significant difference with the pull-through PEG group demonstrating a higher proportion compared to the push GT group (p = 0.0360, Pearson’s Chi-squared test). This difference is attributable to our practice, because in oncohaematological patients we always performed pull-through PEG.

**Table 1:** The occurrence and statistical analysis of underlying medical conditions in the push GT and pull-through PEG groups. The results include the number of cases and their percentages within each group, along with the p-values (from Pearson’s Chi-squared test).

Variable	Group GT	Group PEG	Statistics
Neurological impairment	27 (58.70%)	19 (41.30%)	p value: 0.2969
Metabolic disorders	3 (60.00%)	2 (40.00%)	p value: 1.0000
Tracheostomy	7 (46.67%)	8 (53.33%)	p value: 0.4538
Mechanical ventilation	4 (50.00%)	4 (50.00%)	p value: 1.0000
Oncohaematological patients	0 (0%)	4 (100.00%)	p value: 0.0360
Cardiological conditions	5 (55.56%)	4 (44.44%)	p value: 1.0000
Nephrological impairment	0 (0%)	1 (100.00%)	p value: 0.4500
Immunodeficiency	1 (50.00%)	1 (50.00%)	p value: 1.0000
Others	6 (75.00%)	2 (25.00%)	p value: 0.2759

### Early and late complications in the push GT and pull-through PEG groups

In both groups all gastrostomy formations were successfully performed.

Complications were classified as early (occurring ≤ 7 days after the procedure) or late (occurring > 7 days after the procedure). In addition, complications were categorized as minor or major according to the ESPGHAN Position Paper. The analysis of early and late complications between the push GT and pull-through PEG groups is summarized in Table 2. Regarding minor complications early minor complications occurred only in the GT group, but this was not statistically significant. These complications were infection and fever. Late minor complications were significantly more common in the push GT group, like stomal infection, granulation tissue formation and unplanned removal of the tube. Tube clogging is typical in pull-through PEG. There was no significant difference between the groups regarding early major complications, which were pneumoperitoneum and sepsis in our study. Late major complications occurred in the pull-through PEG group, which were buried bumper and tearing of the the internal bumper.

**Table 2:** The occurrence and statistical analysis of early and late minor and major complications in the push GT and pull-through PEG groups. The results include the number of cases and their percentages within each group, along with the p-values (from Pearson’s Chi-squared test).

Group GT	*N = 33 (%)*	Group PEG	*N = 27 (%)*	Statistics
Early complications		Early complications		
Minor		Minor		p value: 0.19
Stomal infection	1 (3.03)	–		
Fever	1 (3.03)			
Major		Major		p value: 0.68
Pneumoperitoneum	1 (3.03)	Sepsis	1 (3.70)	
Sepsis	1 (3.03)			
Late complications		Late complications		
Minor		Minor		p value: 0.03
Stomal infection	2 (6.06)	Stomal infection	1 (3.70)	
Granulation tissue	4 (12.12)	Tube clogging	1 (3.70)	
Unplanned removal of the tube	4 (12.12)			
Major		Major		p value: 0.01
–		Tearing of the internal bumper	3 (11.11)	
		Buried bumper	2 (7.40)	

## Discussion

To our knowledge, our paper is the first to report percutaneous endoscopic gastrostomy tube placement with a gastropexy device in infants and to investigate its early and late complications.

The classic pull-through PEG method remains the most commonly used technique to provide long-term enteral feeding routes. Minor complications can occur relatively often during PEG placement, and certain major ones are associated also^19,20^.

To evaluate the safety and efficacy of push GT, we retrospectively analysed outcomes comparing push GT and traditional pull-through PEG procedures in patients weighing less than 8.6 kg. We specifically evaluated patient demographics, underlying diseases, as well as early and late complication rates and types.

In particular, patients who underwent push GT were significantly younger and had a lower weight, reflecting a clinical shift towards earlier intervention to meet growing long-term nutritional needs. Despite these demographic differences, basic medical conditions did not differ significantly between the two groups, suggesting that push GT offers a viable early option in this group of patients.

Regarding early minor complications, the push GT group presented one case of peristomal infection and fever. This was not a significant difference between the two groups.

In the push GT group, despite the young age of the patients our peristomal infection rate was lower than previously reported by Jean-Bart et al. (2023), who documented an early infection rate of 3,5% across all age groups^11^. Our findings suggest that, despite the younger age and the potential increased vulnerability of our patient cohort, push GT is associated with a comparable or lower infection rate.

In terms of late minor complications in the GT group we observed late stomal infection in 2 cases (6.06%) Jean Bart et al. showed that with increasing experience, the rate of infection decreased over time^11^.

Granulation tissue formation was observed in 4 cases (12.12%), and the most undesired late minor complication, i.e. unplanned tube removal occurred in 4 cases (12.12%). Granulation tissue formation is a frequent consequence of one-step method, the frequency as a late complication is between 5% and 56,7%, and tube dislodgment is a common problem as early and late complications, although it can be reduced with protocol modifications^9,11,13,14^.

In our study we only observed tube dislodgment as late a complication, between 2 and 16 months after initial placement, which refers more to balloon malfunction than to stoma problems. We think raising parental awareness could help avoid this complication in the future. Early detection and fast tube replacement help to avoid stoma closure. However, in certain cases careful reinsertion is necessary to avoid malpositioning.

Regarding the early major complications, there was one case of systemic infection in both groups. In the push GT group, we observed early sepsis in 1 patient (3.03%) who had ichthyosis and immunodeficiency. Since both conditions are considered risk factors for sepsis, it is not clear whether sepsis was the result of the procedure or not. In the literature, septicaemia is a known but low-rate early major consequence in one-step gastrostomy^11^. We believe that in some circumstances, such as in immunocompromised patients, antibiotic prophylaxis should also be considered for push GT.

In 1 (3.03%) case, we observed pneumoperitoneum, which was caused by excess use of air during insufflation of the stomach. Carbon dioxide (CO_2_) insufflation was not available at that time point. While still observing the patient, the abdominal air was absorbed and passage started. As a result, we think careful air insufflation and suction are obligatory in this age group.

Importantly, we encountered no late major complications such as organ injury, perforation or bleeding in the push GT group, while major complications were observed in the pull-through PEG group.

The clinical implications derived from this study clearly support the use of push GT with gastropexy device as a safer and more comfortable alternative to long-term enteral feeding in infants and young children, especially given the growing demand for early intervention. Stiches may be preferable to T-fasteners, because there are no complications associated with the stiches. Furthermore, despite the young age of our patients, we observed an acceptable safety profile even without routine antibiotic prophylaxis, although selective antibiotic use may be warranted in immunocompromised patients or high-risk cases.

The operative time and feeding after the procedure did not show any significant difference between the two groups.

Regarding costs, if we consider the additional anaesthesia required for pull-through PEG, the one-step method with a gastropexy device is more cost effective.

Several limitations should be acknowledged in our study. First, the retrospective design inherently carries risks of underestimating or overestimating complication rates due to potential incomplete data recording. Second, the sample size was relatively small, which limits the generalisability of our findings. Therefore, prospective multicenter study with larger sample sizes is essential to validate these findings and further explore long-term safety results. The strength of this study is the specialised patient group of the paediatric population, providing focused information on the procedural safety and feasibility in infants.

In conclusion despite its limitations, our study support that push GT with a gastropexy device and stiches is a safe and effective alternative for infants and young children under 10 kg. In our practice, push GT has become the preferred method to perform gastrostomy under 10 kg regardless of the underlying disease. The procedure demonstrates a favourable safety profile with a low incidence of complications, particularly the absence of significant late complications. Future prospective multicenter studies with larger patient cohorts are essential to further substantiate these findings and optimize clinical practice guidelines.

## Data Availability

The datasets used and/or analysed during the current study available from the corresponding author on reasonable request.
